# Supplementary material for: Serum Levels of Soluble Triggering Receptor Expressed on Myeloid Cells-1 Associated with the Severity and Outcome of Acute Ischemic Stroke
Source: J Clin Med. 2020 Dec 26;10(1):61. doi: 10.3390/jcm10010061 (PMC7795761; doi:10.3390/jcm10010061)
Supplement: Supplementary file 1 [file jcm-10-00061-s001.pdf]

Supplementary Material

The exclusion criteria:

1. Patients who fit criteria of intravenous recombinant tissue plasminogen activator (rtPA) thrombolysis, or intra-arterial thrombolysis and mechanical thrombectomy therapies.
2. Transient ischemic attack.
3. Brain injury: intracranial hemorrhage (such as epidural hemorrhage, subdural hemorrhage and subarachnoid hemorrhage) within 6 months.
4. Concurrent renal insufficiency: estimate glomerular filtration rate (eGFR) < 60 ml /min/1.73 m^2.
5. Concurrent hepatic insufficiency: aspartate transaminase (AST), alanine transaminase (ALT) > 200 U/L.
6. Malignancy: patient had history of any cancers.
7. Hematological diseases: such as leukemia, lymphoma or multiple myeloma etc.
8. Immunological or inflammatory diseases: such as the antineutrophil cytoplasmic antibody-associated vasculitis, rheumatoid arthritis, systemic lupus erythematosus, inflammatory bowel disease, acute pancreatitis or chronic obstructive pulmonary disease, etc.
9. Patients on immunomodulatory therapy: patient received immunomodulatory therapy within 6 months.
10. Recent surgery or trauma: recent major surgery or trauma within 6 months.
11. Recent infection: Infection was defined as white blood cell count from blood samples higher than 12000/μL or lower than 4000/μL or with 10% immature (band) form in the emergency room or clinical symptoms of an infection (fever and/or pyuria for urinary tract infection and fever and/or productive cough and radiographic evidence of consolidation for pneumonia).
